# Lessons Learned from a Hybrid Approach to Collecting User Feedback on Coastal Science Products

**DOI:** 10.1007/s00267-026-02530-0

**Published:** 2026-06-12

**Authors:** Zachary Hough Solomon, Dawn M. Kotowicz, Maya K. Hayden

**Affiliations:** 1 Contractor to the U.S. Geological Survey, Reston, VA USA; 2 U.S. Geological Survey, St. Petersburg Coastal and Marine Science Center, St. Petersburg, FL USA; 3 U.S. Geological Survey, Pacific Coastal and Marine Science Center, Santa Cruz, CA USA

**Keywords:** Usability, Contextual inquiry, Environmental decision-making, Development cycle, Actionable science

## Abstract

Science products play a critical role in assisting environmental decision-makers to manage coastal systems; yet there are challenges to developing products that align with needs of end-users. By combining aspects of usability testing and contextual inquiry methods, we describe a hybrid approach for collecting user feedback, especially applicable within the federal government context. This paper presents results of this hybrid testing approach on two of U.S. Geological Survey’s science products, the Coastal Change Likelihood (CCL) and Hazard Exposure Reporting and Analytics (HERA). Both science products synthesize coastal hazard exposure at different geographies and scales. By comparing two science products in different stages of development, we identify common usability issues across both products; the importance of defining a target audience; requested functionalities beyond the products’ scope; and the effect of previous stakeholder engagement on how testers describe technical issues and barriers. Results from user feedback contribute to understanding needs and barriers of coastal decision-makers for science product development. This paper broadens the understanding of environmental decision-makers in the science product development cycle, and how social dimensions can be integrated in project design through iteratively working with end-users and product developers. Further, we present general lessons in usability for development cycles and how these lessons can inform development of actionable science products in coastal management.

## Introduction

### Informational Needs of Coastal Decision-makers

Environmental decision-makers demand relevant information for their specific spatial, temporal, and political contexts (McNie 2007). Coastal decision-makers comprise a diverse group with different needs (Bennett 2019). Coastal systems are complex, integrated ecological and socioeconomic communities, such as fisheries, transportation, and emerging energy infrastructure, which coastal decision-makers must navigate (Hall et al., 2019; Glaeser 2023 p. 135). Over the last 30 years, coastal literature has grown to include research on diverse topics like the development of the blue economy, coastal management zones, and marine protected areas (Bennett 2019; Chen et al. 2023). Given the diversity of users in these complex systems, their information needs vary, and producing science products to meet those needs is an emerging research topic.

Science products can deliver the results of complex coastal science models to decision-makers to support with management considerations in coastal contexts, but many barriers still exist for utilizing these products (Wong-Parodi et al. 2020). The U.S. Global Change Research Program, a federal interagency program, has called for increasing usable and actionable science for decision-makers (Dilling and Lemos 2011), however, Sandifer and Scott (2021) identified the existing need to focus on modeling exposure and vulnerability at different time scales and geographic scales, such as local, county, or state level, because available science products are not sufficient for all types of users. Coastal decision-makers report a gap between the science products that are readily accessible and the extent that the products meet their decision-making needs (Stoltz et al. 2023a; Wong-Parodi et al. 2020; Elko and Briggs 2020; Mostafiz et al. 2022). Adequate science products for mapping sea level rise and other coastal hazards were the biggest challenge reported by Elko and Briggs’ (2020) in their national assessment of 134 coastal decision-makers. This paper addresses the gap that coastal decision-makers face accessing timely relevant and timely science products for responding to coastal hazards.

To pilot our approach, we selected two geospatial science products, produced by the United States Geological Survey (USGS) Coastal and Marine Hazards and Resources Program, one newly released, and another re-released with new functionalities. The first is the Coastal Change Likelihood (CCL) https://geonarrative.usgs.gov/ccl/nps/, a newly released product that provides the user with coastal change predictions on a decadal scale by synthesizing several datasets that describe the coastal landscape and the processes that impact that landscape. The second is the Hazard Exposure Reporting and Analytics (HERA) www.usgs.gov/apps/hera, which provides users estimates of exposure to coastal flooding, coastal groundwater inundation, and shoreline change of a user-defined geographic area. In this paper, we define coastal decision-makers as follows: resource managers and planners, regulators, conservation practitioners, engineers, and researchers from government, industry, and academia that need to describe, map, or assess coastal and marine ecosystems to inform the actions their professional capacities require. We define coastal science products as outputs from scientific research or datasets that are used for planning, management, or understanding coastal hazards.

In this paper, we combine aspects of two user research methods – contextual inquiry and usability testing – to evaluate two science products for technical and development issues specific to each of the science products. In comparing these two science products in different stages of development, we contribute to understanding target end-users’ needs for actionable, usable information, and iterative changes needed to improve the science products on the development side.

### Background: Coastal Science Products - Barriers

The complexity of producing useful science products is based on end-users’ needs, which can be complex and contradictory (Stoltz et al. 2023a). We identify four important barriers in developing science products for coastal decision makers: the geographic fitness of scale, science products appeal, institutional barriers, and the participatory process of design.

Different end-users need different scales of science (Stoltz et al. 2023a), which can be hard to accommodate based on the underlying data (Schumacher et al. 2020). An important factor for tailoring a tool to end-users is the fitness of geographic scale, which, when not aligned with end-users needs, is known as scale mismatch (Guerrero et al. 2013; Lemos et al. 2012). If the science product is developed without the appropriate spatial resolution to address the coastal management problems end-users face, it is not usable (Guerrero et al., 2013).

Different end-users will choose the science product that best factors into their workflow (i.e., a tool designed for everyone is a tool designed for no one) (Gjøsæter et al. 2021; Bamzai-Dodson et al. 2021). Potential end-users are faced with many science products, which can be an overwhelming prospect, making product preference an important consideration in adoption (Raub and Cotti-Rausch 2019; Stoltz et al. 2023a). For example, end-users must find the science product compelling enough to learn to use it compared to the current alternative, and the time it takes to learn, worth the learning curve. Science product developers’ engagement must communicate why to adopt their product versus others (Collini et al. 2022; Stoltz et al. 2023b).

Institutional barriers contribute to the development of science products that fall short in delivering end-users actionable science (Pearman and Cravens 2022). There are institutional factors, such as funding structures for researchers and projects that contribute to these barriers (Matso and Becker 2014). Funding or new priorities can shift focus, interrupting or discontinuing the continuity of stakeholder engagement and user feedback collection on science products (Bamzai-Dodson et al. 2021). A strategic factor is that research agendas may not align with the needs of the science’s end-users (Lemos et al. 2012). When organizations produce coastal science, another established barrier for coastal decision-makers arises; they frequently they do not clearly define a problem for decision-making with their research (Matso and Becker 2014). This organizational disconnection between science production and end-users’ needs can only be addressed through intentional engagement (Matso and Becker 2014). Evaluating end-users’ utilization of actionable, usable knowledge is further complicated by the lack of definition of what developers consider use of the products (Arnott and Lemos 2021). This chain of issues, from research funding to feedback, creates institutional barriers in the development of science products due to insufficient end-user participation in the development process (Stoltz et al. 2023b; Voinov et al. 2016; Haße and Kind 2019). Increasing engagement is complicated by the dynamic in which scientists conducting this work tend to not get recognition for boundary-spanning or stakeholder engagement work within their institutions (Bamzai-Dodson et al. 2021).

Public agencies face unique institutional barriers to addressing public needs for producing science, but various institutional reasons contribute to this usability gap (Pearman and Cravens 2022). Within public agencies, scientists and administrators deal with uncertainty when collecting data or conducting research, particularly when they do not have articulated goals, defined problems, or are uncertain about regulatory constraints (Matso and Becker 2014; Ulibarri 2019). This tension challenges the rationale for producing science for science’s sake, which puts the end-users first (Dilling and Lemos 2011; McNie 2007), and due to the competing interests of agencies and scientists, cutting-edge, “state-of-the-art” research might not benefit end-users (Dilling and Lemos 2011; McNie 2007). Another factor in evaluating the use of science is that agencies and scientists assume the product is used by intended end-users without collecting usage data (Raub and Cotti-Rausch 2019). This paper focuses on integrating usability feedback in the context of the specific challenges federal agencies face in producing government science products.

In addition to institutional barriers to producing actionable science, there are also barriers that prevent end-users from effectively using science products. These established barriers include the participatory process of design; the exclusion of end-users from the development process (Nygård et al. 2020; Schumacher et al. 2020); the inadequate capacity or need for intermediaries (knowledge brokers) between developers and end-users (Chong et al. 2017; Kiem et al. 2014; Palutikof et al., 2019: Laudien et al. 2019); the requirement for improved ‘tool packaging’ rather than simply creating more science products (Kiem et al. 2014; Collini et al. 2021; Palutikof et al. 2019); the differing understandings of uncertainty held by different end-users (Laudien et al. 2019); and diverse perspectives between end-users and scientists (Kiem et al. 2014). Another limitation for end-user access is the maintenance and of science product data infrastructure, where organizations do not provide updates (Nygård et al. 2020; Collini et al. 2022).

### User Research on Decision Support Tools for Coastal Decision-Makers

To document engagement with end-users in previous usability research, we mapped when the engagement occurred in the projects described (See Table 1). The three stages of end-user engagement are described in the following categories: (1) development stage, gathering input prior to or during tool development (2) beta-testing the tool, engagement prior to the official release of the tool, (3) and post-launch of the tool, engagement after release of the tool. Of the five papers that focused on a singular tool, two papers engaged with end-users in all three stages (Collini et al. 2022; Palutikof et al. 2019). Goble et al. (2019) engaged with their end-users in the development stage and beta-testing stage, while Collini et al. (2021) and Laudien et al. (2019) engaged with their end-users’ post launch in their projects. This rough delineation of development stages was only mapped for papers that focused on a single tool, papers that focused on more than one was not categorized. The papers that examined multiple decision support tools did so to learn broader usability lessons and inform science-policy gaps (Bogatinoska et al. 2022; Chong et al. 2017; Hart et al. 2022; Kiem et al. 2014; Nygård et al. 2020; Schumacher et al. 2020). We used the term “decision support tool” in the keyword search to conduct the review (and it is used throughout 1.3), whereas “science product” is used throughout the paper as the broader term for research-derived information outputs.

**Table 1:** Papers reviewed in literature review

Author	Stages of engagement	Methods
Bogatinoska et al. (2022)	N/A-	Interviews, webinars, workshops
Collini et al. (2021)	3	Interviews, usage analytics
Collini et al. (2022)	1,2,3	Focus group, Survey, webinars, workshops
Chong et al. (2017)	1,2	interviews
Goble et al. (2019)	N/A	Survey
Hart et al. (2022) Kiem et al. (2014)	N/A N/A	Survey Survey, workshop
Laudien et al. (2019)	3	Interviews
Nygård et al. (2020) Palutikof et al. (2019)	N/A 1,2,3	Survey Interviews, Survey, Workshops
Schumacher et al. (2020)	N/A	Survey

Stages of engagement are defined as follows: (1) development stage, gathering input prior to or during tool development, (2) beta-testing, engagement prior to the official release of the tool, and (3) post-launch, engagement after the tool’s release, N/A indicates papers focusing on multiple tools.

In reviewing these papers, which include engagement in different stages of tool development, we document how other researchers are engaging with users through different methods and in different stages of the development cycle. These papers employ surveys (e.g., paper questionnaires, web surveys) most commonly to understand usability and user experience.

### Usability Testing and Contextual Inquiry Methods as a Hybrid Approach

Usability testing employs preformatted questions or tasks to test the functionality of specific aspects of a product. Contextual inquiry, another method of evaluating user experience, focused on understanding users in their natural setting, and was developed to allow users to direct researchers to what they identify as important (Holzblatt and Beyer 2014, p 3.). Technology has changed the way people work and their work settings, however, contextual inquiry adjusts to account for users’ workflow in product design in the twenty-first century (Holtzblatt and Beyer 2014).

Contextual inquiry and usability testing were combined into a hybrid approach due to the goals of gathering timely feedback on science products, identifying technical issues, and exploring users’ perceptions of the products’ applicability to their work. Implementing usability evaluation is feasible because the evidence shows that a relatively small sample of five (5) participants can yield 85% of significant issues (Nielsen and Landauer 1993). There are differences in these methodologies, however, they can be complementary, whereas, contextual inquiry is emergent, and usability testing is fixed. Holtzblatt and Beyer (2014 p. 2) articulates that any “usability testing…or any data gathering technique with fixed questions or predetermined tasks -- takes the user out of the context of his or her life.” Rosenbaum (2000) details that usability testing and contextual inquiry hybrid methods are possible when clearly articulating the research goals (Rosenbaum 2000). Kronberg (2020) found that usability testing versus contextual inquiry yielded different user information on the same product, this rationale of gathering multiple types of information informed the test design. This hybrid approach can be used to gather feedback on science products utilizing the strengths that both methods provide to assist public agencies, such as the USGS, in evaluating science product usability in a timely way (Table 2).

**Table 2:** Hazards Exposure Reporting Analytics (HERA) and Climate Change Likelihood (CCL) Testers’ Affiliation

Science Product	Tester Affiliation
HERA	Sea Grant
HERA	Sea Grant
HERA	Private industry
HERA	Municipal Government
HERA	State Regulatory Agency
CCL	National Park Service
CCL	National Park Service
CCL	US Fish and Wildlife Service
CCL	University
CCL	University
CCL	University extension

## Methods

### Test Design

The combination of both contextual inquiry and usability testing methods, hereafter called the hybrid approach, was developed to collect information on ways of improving the science products’ performance and to learn about testers’ perspectives and workflows. The protocols for the two products were developed using principles from the six-step process outlined by Rubin et al. (2008) to evaluate their usability. We recognize that both products have different methodological underpinnings and history of use, however, we used the same methods – though different protocols – to gather feedback relevant and actionable for development of both products – HERA and CCL. The authors worked with both science product development teams and stakeholder engagement specialists to identify the most relevant functionalities to test and to identify potential testers recruitment. Testers were recruited via email sent either by a product development team member or stakeholder engagement specialist familiar with the potential tester, which introduced the author and explained the testing process. Prior to beginning the testing, each protocol was initially pre-tested with staff from the USGS.

Each protocol included an allocated amount of time for participants to “free scroll” (e.g., explore the product site), interpretive questions on parts of the product site, predetermined usability tasks, and follow-up questions about motivations and likelihood to use the product (see Supplemental Materials). Testers were instructed to think-aloud during testing (Boren and Ramsey 2000). CCL’s protocol was designed to last approximately 30 min and was created in October 2023. Six testing sessions were conducted between November 6 and November 13, 2023. For the HERA evaluation, two protocols were developed to cover both the Coastal Groundwater and Shoreline Change functionalities of the HERA. The protocols were developed in October of 2023, and the five testing sessions were conducted between December 4 and December 15, 2023. The protocols for HERA were initially designed to test additional functionalities but were pared down after pre-testing to fit the intended duration of 45 min. A total of 11 testers participated.

With participant permission, interviews were audio-recorded through virtual meetings with Microsoft Teams. The online setting for testing was chosen to be able to include interviewees from multiple states. Interviews ranged from 27 to 46 min in length, for 417 total min of data. Testers were affiliated with three different federal agencies, one state commission, three universities, one private industry, and one municipal government.

**Fig. 1:**
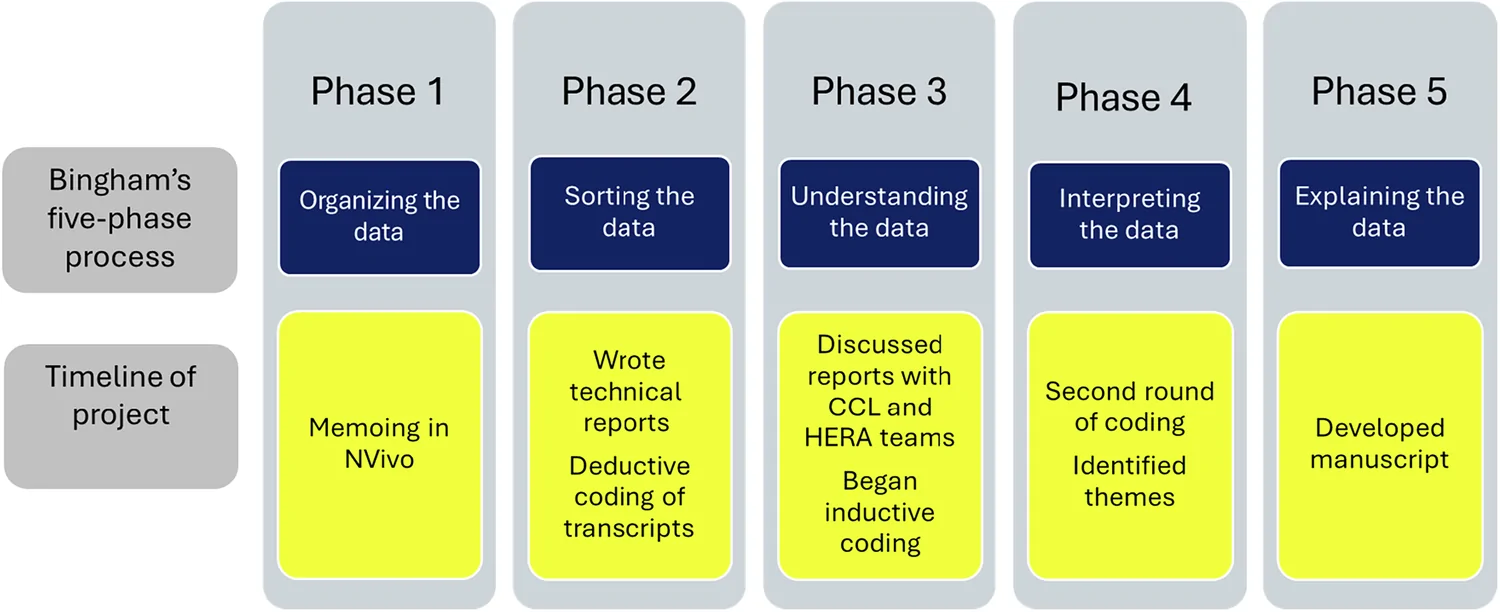
Five phases of project development

Audio files were transcribed through Microsoft Teams, then uploaded as a Word document to the qualitative data analysis software NVivo for cleaning and preparation for analysis using NVivo Release 1.7 (Lumivero; Denver, CO). Memos were employed in NVivo to develop codes; memo writing is the process of reflexively describing real-time notes and events to increase the context of the coding process (Saldaña 2013). Coding is the first step of this analysis, which assigns descriptive labels to the data to extract thematic ideas (Saldaña 2013; Gibbs 2007). Bingham’s (2023) five-phase process of data analysis was used to analyse the interview transcripts throughout the project’s cycle (Fig. 1).

In Phase 1, we developed memos from the transcripts in NVivo. In Phase 2, we analysed transcripts, beginning to develop technical reports for the product development teams. We identified deductive codes, such as select technical problems on the navigation and user function (Hough Solomon 2025). In phase 3, through meetings with both science products’ development teams, we disseminated early findings about technical issues revealed during the testing. We finished the preformatted deductive codes and began to address the inductive analysis, for example, drafting emerging codes from technical problems to inductively summarize feedback about questions such as motivation to use the product (Hough Solomon 2025). In phase 4, we proceeded with secondary coding, incorporating developer perspective and refining inductive emergent codes. This allowed us to identify specific challenges for each product (e.g., problems with navigation) and broader issues for the overall project (e.g., importance of defining a target audience). In phase 5, we synthesized results from multiple iterative rounds of reporting findings to the science product development team along with the constant comparison method Charmaz (2014) and inductive and deductive reasoning to identify broader themes (Bingham and Wikowsky 2021).

### Findings: Thematic Results

Based on the end-user feedback collected from this research, four main themes emerged from the coding process: (1) the hybrid testing approach revealed common usability issues across both products; (2) the importance of considering a target audience in user testing and product development; (3) testers requested features outside the product’s functionalities; and (4) the impact of previous stakeholder engagement on user perspectives around using these products.

### Hybrid Testing Reveals Common Usability Issues Across Two Science Products

Despite differences in the function of the two science products and the types of end-users tested, the usability evaluation revealed several common technical issues that testers described as barriers to using the products. By analysing the two products together, we detected common issues about accessibility (e.g., color blindness, color scales/bins, display of boundaries), navigation (e.g. lack of zoom-to-function, searchability), and help support for troubleshooting in the products. For instance, one CCL tester stressed that the transparency needed to be addressed for one of the outputs during the usability evaluation. In a follow-up question at the end, the tester stressed that the lack of that function was critical to adopting the product, “You need to fix this” (CCL Tester, 11-7-2023). These technical problems provided reasoning for why users would or would not use the product based on the user experience. The technical issues were identified both through responses to pre-formatted questions testing specific features, and by users identifying barriers in the product’s interface.

In the evaluations for both HERA and CCL, the use of “free-scrolling” revealed baseline information about the science products and their perspectives. In the HERA testing, users were asked to describe their perception of the products’ intended use at both the beginning of the testing, and again at its conclusion. The repetition of this question helped clarify if and where there was confusion about how testers perceived the product’s intended use and their motivations to utilize it for decision making. By querying testers after a set amount of time of “free scrolling” while observing the tester “thinking-aloud,” the tester was asked to synthesize their understanding of the product before moving on to usability tasks – the use of contextual inquiry in the beginning and in the end identified issues that might not have been discovered in the usability evaluation. One finding was that the HERA testers defined a broad range of interpretations of its intended use in the outset of the testing and all tester responses changed after using the product in the predetermined usability tasks. In the CCL testing, the use of contextual inquiry was used to explore the geonarrative and website where testers “free-scrolled” and were prompted to “think aloud” as well as responding to reacting to specific slides in the geonarrative. This portion was structured in the protocol prior to the usability tasks and uncovered baseline information from the testers to understand their motivations. These sections provided information to better refine the website about the underlying datasets and model in the outputs.

The use of these techniques in testing both products helped to understand the testers’ previous knowledge of both products and their motivations to use the science products. Through the responses in the HERA testing, it revealed differences in how the testers defined its purpose and intended use. This suggests that the HERA site could attract more users if its function were more clearly communicated on its introductory page. The testers’ responses about the science product’s function were based on their previous knowledge; the differing range of interpretation, highlight an opportunity for the science product’s site to better communicate its purpose to target users. In the CCL testing, the “free scroll” testers provided useful information about how the broader site communicated the underlying data and model as an issue. This user feedback for both science products was communicated to the development team to consider for updates to both development teams.

### Insights for CCL and HERA: Target Audiences Emphasize Importance of Scale

CCL’s target audience reported that they were confident in utilizing the product for making management decisions, citing the appropriateness of scale for their work. CCL, a beta-version release, was initially developed as a decision-making science product for the National Park Service, the science product’s target audience, but the development team identified other additional users to test. A difference emerged between testers who identified themselves as researchers versus decision-makers in terms of their interest in using the science product for their work, with 4 out of 6 CCL testers indicating that they would use the product in some capacity if the data were available. Before the usability evaluation, testers self-identified as either primarily researchers or decision-makers in their work. The latter group was the target audience for the product, specifically the National Park Service. Both National Park Service testers reported interest in using it for their work, and one reported they are already planning on using the CCL for managing salt marsh habitat: “We’re already looking to use it for some strict-structured decision making” (CCL Tester, 11-13-2023). The two researchers in the testing reported the product did not accomplish the scale of localized information and accuracy for the scope of their work. The researcher feedback indicates that the scale mismatch is likely due to trade-offs with the product design, which was tailored for decision-makers. This variation between the two sets of testers reflects that this is a barrier to using the science product.

The HERA product showed greater variation in responses to interest in using it due to a broader range of end-users (e.g., private industry, municipal planners, state regulators) and a less-defined target audience. For example, a local city planner was not convinced that the assumptions of the model were correct for their management areas. “We can’t use it because some of the starting assumptions aren’t accurate” (HERA Tester, 12-3-2023). Another tester mentioned that they would potentially use the product if it was available for their geography for looking at “15 different counties” (HERA Tester, 12-4-2023). The target end-user for HERA is less defined compared to the CCL science product, which is narrowed but attempts to be applicable to a broad set of users. The greater variation in responses from HERA testers could be due to the many types of users we tested.

### Testers Requested Functionalities Not Provided in the Science Product

User feedback for both CCL and HERA science products highlighted a strong demand for greater data precision and other functionalities outside. During testing, we collected information on potential functionalities beyond the current scope of the science products to understand what testers wanted. Two of CCL testers requested the integration of utilities and other infrastructure data into the science product, which is beyond the scope of the current beta version. Notably, CCL researchers, who were not the target end-users, requested different features outside of the scope of the science product’s functionalities. CCL testers also inquired about precision to a finer scale. Through interpretive questions and the time spent free-scrolling, testers requested more precise data than what were available in both CCL geonarrative and HERA products. In the HERA testing for the Coastal Groundwater and Shoreline Change functionalities, multiple testers requested discrete exposure analytics (whole numbers) at the census block-level, alternatively to averaged impacts for different scenarios at the block level. One tester described that the block had a range of exposure from sea level rise but wanted an exact number of units exposed (occupied housing units was the selected asset), “would I be able to click on this block and get the precise number between 69 and 269” (HERA Tester, 12-7-2023). This user feedback was communicated to the technical staff on the project team when we reported the technical issues for planning updates to the product.

While communicating initial feedback to the HERA development team, they highlighted that block-level precision could obscure the accuracy of the datasets and the represented science, indicating that limits to feedback must be intentional. Developers explained that based on the dataset underlying the product, setting the number to an exact number of impacted housing units would be less accurate, and that range would challenge the product’s functionality. They explained that said updates would conflict with the underlying data constraints and incorporating them could make the outputs less accurate. This emphasis in testing for greater data precision and added functionalities can help understand the requirements of target users in future stakeholder engagement.

### Previous Engagements Influenced User Testing

The interview data reflected the extent of previous stakeholder engagement’s influence on user testing across the two products. All five testers reported exposure to HERA or prior training with COSMOS, which uses the underlying dataset, for HERA. Tester’s familiarity with COSMOS likely contributed to their high investment in using HERA despite encountering more bugs, crashes, and delayed responses from the server as compared to the CCL testing. A HERA tester who was familiar with COSMOS was interested in using the product for their work, even after encountering many technical problems. They stated: “There is pretty big lag, which I didn’t recognize as a lag at first…so, then I just kept pressing the button over and over again.” (HERA, 12-5-2023). We attribute users’ familiarity with COSMOS to COSMOS’s development team’s stakeholder engagement efforts during development of that product. This is supported by the results from the 2023 California Coastal Adaptation Needs Assessment where coastal decision-makers turn to COSMOS as their primary source for sea level rise and flood information and due to their familiarity with it (Grifman et al. 2025, Pg. 38-39). In contrast, CCL testers, using a newer beta-stage product, were more concerned with data questions rather than user experience, (e.g., questions about the underlying dataset, the implications for different scenarios, and statistical questions about the science product layers). For example, one of the testers was distracted from the usability tasks because they were focused on how the model behind the output functioned.

## Discussion

### The Hybrid Approach Allowed User Feedback on Specific Functionalities and Broader Concerns About Science Products

The project’s hybrid approach to data collection allowed us to identify priority problems to be fixed based on both the functionalities identified by the product developers and the issues identified by the end-user testers. Drawing from usability testing methods, we were able to test specific functions identified by the development teams for each of the products, such as navigation and the ability to produce outputs, and we were able to report back user experience on those specific usability issues. Integration of contextual inquiry into testing revealed additional issues to emerge, including end-users’ understanding of the product’s purpose, enabling them to define for themselves the most important aspects of the product, and to identify concerns outside the scope of the preformatted functionalities. By learning about the usability experience and user defined preferences for science products, this information can be prioritized for future iterations of the product to better address the needs of end-users (Lemos et al. 2012). The information collected via this research, across two coastal science products, such as the need for better navigation and challenges surrounding scale mismatch, can be considered along with existing information to tailoring products in-situ for environmental end-users (Stoltz et al. 2023b).

The hybrid approach used to gather end-user feedback in this study provides an example of a feasible, efficient way to gather usability information from potential users on science products produced by a federal agency. The 1995 Paperwork Reduction Act (PRA) requires extensive approvals for federal agencies to collect information from the public (44 U.S. Code § 3501; Shapiro 2013), increasing budgets and extending timelines for data collection. However, Executive Order 14058 (2021) and the November 21, 2024, Memorandum provide an opportunity for agencies to conduct usability testing that employs “empirical methods” to improve the delivery of customer experience (86 FR 71357; Memorandum 2024). This mandate emphasizes “customer”, or user experience, which includes research and stakeholder engagement. We highlight that using a hybrid usability testing and contextual inquiry approach can systematically collect this information for specific products and gain a broader understanding of the perspectives of the target audience while maintaining compliance with federal policy. This type of research can identify barriers that end-users face in using science products by iteratively learning about end-users and working toward improving existing products or better development of new science products (Stoltz et al. 2023b).

### The Importance of Tailoring Science Products for a Target Audience in the Development Cycle

We identified the importance of considering a target audience in science product development for these two science products. For CCL, testers represented a narrowly defined target audience focused on roles for which the product was designed. For HERA, identified testers represented a broader diversity of roles. The testers reported that CCL’s produced outputs at an appropriate scale to meet their decision-making needs. For HERA testers, some participants reported that aspects of the product met their needs but that the information presented did not match the geographic scale at which they had to make decisions. The scale mismatch with some of the HERA testers can be partially attributed to the diversity in testers. HERA’s development team could better meet end-user needs by narrowing down the target audience to prioritize improvements for a smaller set of end-users. Conversely, the lack of reported problems with scale and spatial resolution can be linked to the more narrowly defined target audience for CCL. This contrast in feedback between the two products illustrates the strength of comparing multiple products together, which can aid in identifying barriers that might not be revealed otherwise. These findings, collectively, suggest the importance of focusing on a specific target audience and developing science products to adequately meet their unique needs for exposure, vulnerability, and adaptation.

Based upon our findings, we identified the importance of engaging with our end-users at all stages of developing science products. By evaluating two science products with differing levels of stakeholder engagement simultaneously, we identified that early-stage engagement can yield usability feedback that help developers better understand needs of the target audience. HERA was launched several years ago, with periodic and consistent engagement between potential end-users and researchers; the research presented here tested new functionalities of the existing product. CCL is a new science product being released for the first time; CCL testers were not familiar with the product, did not report past engagement efforts and had less patience for the product; however, CCL testers reported that the science product fit their needs for decision-making. For CCL, more frequently engaging with end-users about the use, purpose, and goals of the product, could lead to more usage and allow developers to gain a greater understanding of users’ needs in implementing upgrades and additional functionalities (Lemos et al. 2012). HERA’s testers had greater patience for lag time and bugs in the testing but were more familiar with the product, given their experience with past engagements (Grifman et al. 2025). Iterative improvements that prioritize the updates identified from this testing can be used to continue to engage with end-users and improve the usability of products for their target audience(s) (Bamzai-Dodson et al. 2021). Furthermore, iterative engagement with these audiences can help both increase the usability of these products for future updates, and development of other science products, and contribute to general knowledge about coastal decision-makers that utilize these two products (Lemos et al. 2012; Stoltz et al. 2023b). Future usability work should continue to draw on the findings from literature about end-user participation in product development at all stages to reduce barriers (Collini et al. 2022; Haße and Kind 2019; Voinov et al. 2016).

### Bridging Between Science and Decision-makers: A Two-way Street of Learning

The approach presented here illustrates how social dimensions, in this case of end-users’ perspectives, can be considered along with physical science in usability research. First, by working as an intermediary between end-users and development teams, we were able to understand the development teams’ perspectives on how they best balance user requests with the constraints of the underlying data when deciding to integrate end-users’ requests for changes (Stoltz et al. 2023b; Stempel et al. 2025, pg. 269). In the meetings with the development teams, we discussed the challenges of integrating end-users’ requests for greater functionalities into the existing products and developers’ concern that certain requested updates can challenge the functionality, accuracy, and original goals of the science product. Although we did not explicitly ask end-users if they wanted other features, utilizing contextual inquiry results in a collection of requests for the product to better suit end-users’ workflow. A broader lesson for selecting this method is to intentionally consider at what stage of product development to consult with test users, and to assess the development team’s capacity to implement changes to the product, prior to collecting information (Lemos et al. 2012; Matso 2012). These considerations highlight the importance of boundary spanning or two-way stakeholder engagement that can work at this interface between product developers and end-users, to better meet the needs of end-users while considering the challenges of technical staff to adapt to users’ needs (Lemos et al. 2012; Collini et al. 2022; Bamzai-Dodson et al. 2021; Stempel et al. 2025).

End-users reported high interest in providing feedback and there was no indication of usability burnout, documented in other end-user focused research, during this project (Kiem and Austin 2013). HERA testers were eager to participate in the study and easy to recruit, which is likely due to the targeted recruitment process and previous stakeholder engagement (Wang et al. 2024). We observed more hesitancy to participate among end-users recruited for the CCL testing, which was likely because they were less familiar with the science product. Although we experienced hesitancy to participate in the study, we did not find research fatigue during user evaluations which has been documented as a significant issue for user research (Kiem and Austin 2013; Kiem et al. 2014). The test design’s interpretive questions allowed the user to focus on specific, pertinent issues they face in their role; the greater control to direct parts of the session empowered the tester and provided rich feedback (Wang et al. 2024). The willingness of participants to contribute their unique perspective while testing specific functions and aspects of these products illustrates a strength of the hybrid approach to collecting usability information (Rosenbaum 2000). Moreover, by working with end-users for evaluating the usability of physical science products, usability work can complete an iterative cycle (i.e., feedback informs developers’ future updates), to work towards more effective science delivery (Dilling and Lemos 2011; Wong-Parodi et al 2020; Stoltz et al. 2023a; Stoltz et al. 2023b).

### Learning About Coastal Decision-makers Can Improve Actionable Science Products

Social sciences can help with understanding challenges for actionable science in government and academia (Dilling and Lemos 2011). Applying social science to usability evaluation can address some of the knowledge gaps between product developers and target end-users, which can inform actionable science product design that better meets the needs of coastal end-users. Iterative and continuous two-way engagement with coastal end-users can result in information that can be collectively analyzed to form a greater understanding of coastal decision-makers. Considering these users’ perspectives during research design can result in science questions that combine cutting edge physical science with decision-maker concerns at the outset, resulting in more actionable information that can then be developed into science products for researchers and decision-makers alike (Kiem et al. 2014; Matso 2012; Arnott and Lemos 2021; Stoltz et al. 2023a).

This study has limitations due to balance of complying with federal agency regulatory landscape and delivering rapid results with a limited sample size, which might have limited the depth of insights. However, this work illustrated the feasibility of conducting work compliant with the regulatory framework under which researchers in public agencies operate. While the sample was limited, usability research has documented the strength of small samples in identifying major barriers for end-users (Nielsen and Landauer 1993). The sample testers were also admittedly non-biased as the products to be tested were not designed for the general public but for environmental decision-makers with a baseline of science and place-specific information. These factors should be considered when interpreting the study’s findings. It should be noted that this study does not examine trust in the products tested, but rather their ability to meet decision-makers’ needs.

## Conclusion

We present the results of testing the usability of the two USGS science products, HERA and CCL, individually and collectively. For this project, we employ a hybrid approach to better understand end-users’ needs and barriers to utilizing the science products. Our findings highlight common issues across science products: the importance of gathering user feedback in the development and evaluation of science products; testers requested features outside the science product’s functionalities; and the impact of previous stakeholder engagement on end-user perspectives when using these science products. We describe how this work can be replicated to inform future usability work and draw broader lessons about conducting usability in coastal systems. Furthermore, the study demonstrates the feasibility of conducting usability testing within the federal government’s regulations and aims to inform future research approaches in that context.

Building off this work, research focused on further developing the hybrid usability and contextual inquiry approach to test multiple science products at different stages can provide a broader understanding of coastal decision-makers information needs (Laudien et al. 2019). Additionally, analysing back-end use metrics along with end-user feedback can provide a greater understanding of the effectiveness of the user experience (Laudien et al. 2019; Patutifik 2018; Collini et al. 2021). This information can be used to further our understanding of coastal decision-makers, thereby improving the actionability of our science, and its resulting products.

## Supplementary information

Supplemental information

## Data Availability

The frequency data for the analysis of the themes are published in this data release: https://doi.org/10.5066/P13HREQN.
