# Supplementary material for: Lessons Learned from a Hybrid Approach to Collecting User Feedback on Coastal Science Products
Source: Environ Manage. 2026 Jun 12;76(6):214. doi: 10.1007/s00267-026-02530-0 (PMC13680294; doi:10.1007/s00267-026-02530-0)
Supplement: Supplementary file 1 [file 267_2026_2530_MOESM1_ESM.docx]

# Supplemental

**CCL Usability Protocol**

**Brief Introduction** [1 minute]

Tester will introduce themself and describe the structure of the interview. Thank you for joining me to test our science product. We scheduled this interview for 30 minutes but may not take all that time. Is it all right if we record this interview for note-taking purposes? Thank you.

**[Press record]**

**Pre-interview questions:**

Answer before we begin the testing [2 minutes]

1. What is your current role at your organization? What previous experience did you have before beginning this position?
2. What geography are you working on, and from where?
3. Who do you consider the constituents or stakeholders you collaborate with or work for?
4. How do you define the role of your work? For instance, do you think of yourself as a researcher, decision-maker, or facilitator?

**Interview Script**

**Project Description** [2 minutes]**:**

In a moment, I’m going to put a link in the chat to our new website. You may know some information about the site and the Coastal Change Likelihood assessment, but we don’t want to give you too much background about it because we want to see how you use it. We’d like your feedback on anything from the way it looks to the way it functions. To gather this information from you, we’d like to spend the rest of our time doing what is referred to as a contextual inquiry. To do this, we ask that you pull up the link from the chat into a browser and share your screen with us so we can see how you interact with the site. As you explore the site, we ask that you talk out loud and walk me through what you’re thinking, and doing, and why. The goal of this discussion is to identify ways to improve users’ experience, so please do not hesitate to tell us any issues or things you think could make this a better site.

**[Put website link in chat]**

[**https://geonarrative.usgs.gov/ccl/**](https://geonarrative.usgs.gov/ccl/)

Before we start, do you have any feedback or first impressions on this first page?

What do you think the CCL assessment can be used to do?

**Tasks**

**Free Scroll** [4 minutes: 2 minutes + 1 minute for explanation and navigation to website + 1 minutes for questions]:

To begin, please open the link in the chat; it will launch you to the CCL tool. The CCL homepage explains the history of the tool, its data, and its uses.  After the page has loaded, take 5 minutes to move through the scroll. Talk through what you are seeing and any immediate questions or comments that you have about what you are seeing or expect to see.

- Did they complete the entire scroll:
- Did they use the Navigation bar/main menu
- Open the data report

**Slide Interpretation**

Next, I am going to direct you to specific slides in the scroll and ask you to talk through what you are seeing. Acknowledge that this might be repetitive following the free scroll.

1. FABRIC - what is the coast made of?

Please click on the main menu on the “Fabric” icon, then proceed to read through to the bottom where the four images are.

**[Paste into chat]**

2. Please click on the main menu on the “Hazard” icon, then scroll past the first word block, and read the section under “What data are used to create the HAZARDS?” and stop where “How do we create the HAZARD datasets?” begins

**[Paste into chat]**

3. Please navigate to main menu and click on the “Explore CCL” icon, then scroll down to the “A Closer look” and read the section underneath until you get to the “Questions” sections.

**[Paste into chat]**

**Explore the Geonarrative** [10 minutes]

Please navigate to the geonarrative through the Explore CCL page to launch. The interactive Map outlines five National Parks on the Atlantic Ocean, and the CCL is composed of Hazard Impact types and Coastal Change Likelihood values.

**[Alternatively, if this person works with the Park Service - of the 5 example Parks, I will direct them to identify a location within their Park’s boundaries that they are familiar with. I will direct them to locate it and describe the context of this area.]**

1. The first task is to navigate to Thompson Island in Acadia National Park, which connects the mainland to Mount Desert Island in Acadia National Park in the geonarrative. You should zoom to 2,000 feet. The airport should be visible in your map's top or top-right corner.

**[*Time Check*]**

**[Paste this into the chat after you read out loud. If they cannot find it at first, describe that it is the small green triangle in the most northwest of the park boundary. This sits atop Mount Desert Island – the landform that resembles a pair of lungs.]**

2. After you locate the area, please turn off the **Hazard Type** and keep the **Max CCL** on. Then switch, turn the **Max CCL** off, and turn the **Hazard Type** off. Then, turn both the **Max CCL** and **Hazard Type** back on.

**[Paste this into the chat after you read out loud.]**

3. Lastly, can you describe the Max CCL values of the largest polygons on either side of the road on Thompson Island? Zoom to 1,000 feet and interpret the legend.

**[*Time Check*]**

**Questions** [if 25 minutes have elapsed, check to see if they can go slightly over the allotted 30 minutes, or skip directly to the wrap up questions]:

1.) Do the Max CCL values surprise you at this specific place?

2.) Based on what you see at Thompson Island Information Center, note that there are public restrooms and picnic areas on the grassy cleared site on the eastern part of the island. How might you think about adaptation at this site, and what changes might you resist, or changes that you would monitor, or infrastructure that you would move or evacuate?

**Wrap Up** [5 minutes]

1. Do you think that you would use this data in your current work? If so, how? If not, why not?
2. Do you think that you would use this site with your collaborators? If not, why not?
3. What motivates you or interests you to use this tool in the future?
4. Does this tool seem more suited for research or decision-making? And can you provide an example of what parts make you think so.

Thank you for your time. We will be speaking with several other intended users and will compile the results of all of the interviews. If you have any further questions or comments, please reach out to me at <your email here>.

**HERA Groundwater Inundation Usability Protocol**

**Introduction** [2 minutes]**:**

Briefly introduce yourself, the product, and the structure of the interview. Thank you for joining me to test the HERA tool. We scheduled this interview for 30-45 minutes, but it may not take all that time. Is it alright if we record this interview for internal note-taking purposes? Thank you.

**[Press record]**

**Pre-test questions** [1.5]:

Before we get started with the usability testing I would like to get a little bit of background information. These questions should only take a minute or two to answer before we begin the testing.

1. What is your current role at your organization?
2. What geography are you working on, and from where?
3. Who do you think of as the constituents or stakeholders that you collaborate with or work for?
4. How do you define the role of your work? For instance, do you think of yourself as a researcher, decision maker, or facilitator?
5. What is your level of familiarity with HERA overall?
a. None: I’ve never heard of it
b. Very Low: I’ve heard of it, or seen it presented, but never used it myself
c. Low: I’ve heard of it, and tried it out at least once
d. Moderate: I am an occasional user (e.g., a few times a year, or periodically as a project might require)
e. Moderate-High: I’m a frequent user (e.g., a few times a month)
f. High: I use it all the time (e.g., weekly)
6. Ask same “level of familiarity” with the specific tool being tested (Groundwater or Shoreline Change)
7. What is your level of familiarity with the Coastal Storm Modeling System (CoSMoS), which provides the hazard exposure maps that underlie HERA?
a. None: I’ve never heard of it
b. Aware: I’ve heard of it, or seen it used, but I’ve never worked on a project where it was used
c. Familiar: I’ve worked on or reviewed a project/effort that has used the CoSMoS data or Our Coast Our Future viewer
d. Well-versed: I’ve worked on or reviewed multiple projects/efforts that have used the CoSMoS data or Our Coast Our Future viewer
e. Power user: I use it frequently (e.g., monthly) either directly with the GIS data or via the Our Coast Our Future viewer

**Interview Script**

In a moment I’m going to put a link in the chat to our website. You may know some information about the site and the Hazards Exposure and Reporting Analytics (HERA) work, but we don’t want to give you too much background about it because we want to see how you use it. We’d like your feedback on anything from the way it looks to the way it functions. To gather this information from you, we’d like to spend the rest of our time doing what is referred to as a contextual inquiry. To do this, we ask that you pull up the link from the chat into a browser and share your screen with us so we can see how you interact with the site. As you explore the site, we ask that you talk out loud and walk me through what you’re thinking, and doing, and why. The goal of this discussion is to identify ways to improve users’ experience, so please do not hesitate to tell us any issues or things you think could make this a better site. Remember that this is NOT a test of your knowledge or abilities, but of the strengths and weaknesses of the HERA product design and delivery, so please don’t worry if you get stuck somewhere or if you “break” something.

**[Put website link in chat]**

**https://www.usgs.gov/apps/hera/groundwaterTool.php**

Before we start, do you have any feedback or first impressions on this first page?

**Free scroll – 3 minutes**

What do you think this tool does?

**Usability Tasks**

I am going to provide you with directions to complete a brief analysis with the HERA tool. The directions that I will provide will not be exhaustive so that we can test the user experience of the HERA’s design, not the user’s, or your, abilities. This tool analyzes the impacts from sea level rise on groundwater depths through inundation.  By mapping the groundwater hazards in different scenarios, HERA informs the exposure of assets, ecology, and communities. The end goal of this task is to determine the exposure to rising groundwater hazards of improved lands in Santa Cruz County under the following conditions.

**Scenario #1:** [10 minutes]

- First, try to view results of groundwater hazard exposure for Improved lands (asset) for the communities of Aptos, California and Santa Cruz, California, both located in Santa Cruz County. Please view the projected exposure of these communities when Sea Level Rise is 6.6 feet the Groundwater depth is cumulatively Shallower than **16.4 feet,** and the geology is **More Permeable**. If you can complete this, please view the results in the dashboard and interpret the results.

**Scenario #2** [5 minutes ]

- Next (after they completed the initial task ), can you set the geology to **Moderate Permeability** from **More Permeable** ? What changes are there to Aptos and Santa Cruz in terms of the **Amount of Improved Parcel Value (Dollars) in the Hazard Zone?** If you can complete this, please view the results in the dashboard and interpret the results.

**Scenario #3** [5 minutes]

- Next, clear your selections in the panel. Can you determine the **Amount of Improved Parcel Value (Dollars) in the Hazard Zone** for Santa Cruz County as a whole? Please determine that based on these adjustments where Seal Level Rise is set to **6.6 feet**, Groundwater Depth is set to cumulative **16.4** feet, and Groundwater Geology is set at **Moderate Permeability**. Then see what the difference is at the county level where Groundwater Depth is set at **6.6 feet** without clearing any of the selections**.** If you can complete this, please view the results in the dashboard and interpret the results.

**Wrap Up** [questions if there is time, est. 2 minutes]

1. Do you think that you would use this data in your current work if it was available for where you are working? If so, how? If not, why not?
2. If it was available, do you think that you would use this site with your constituencies or partners? If not, why not?
3. What kinds of platforms/hardware would you use it on? Such as accessing HERA via desktop, laptop, or tablet.

Thank you for your time. We will be speaking with several other intended users and will compile the results of all of the interviews. If you have any further questions or comments, please reach out to me at <your email here>.

**Troubleshooting and Questions to Ask**

**Places**

- *If they are struggling to select the correct geography* In the tool, please go to the panel on the left and select  the dropdown triangle for California under **Places,** then select the dropdown for Santa Cruz County. A border should have popped-up around the county.  After that, follow the drop-down under Santa Cruz County, and navigate to the Communities level, and then select Aptos and Santa Cruz in the drop-down. If they get stuck (or they cannot find the selection direct them to the ⓘ).

**Questions:**

- Can you describe what you are seeing on the map layers in terms of the colors, shapes, and contrast. And is it easy to decipher the boundaries and layover of the polygons?
- Is it lagging at all while navigating between selecting different communities or zooming-in or out?

**Groundwater Hazard**

- 1) *If they get stuck selecting the correct sea level rise, groundwater depths (or when they cannot complete the correct selection, direct them to the ⓘ)*. “Can you briefly talk through if this makes sense?”

**Mini-task:**

- “Can you navigate to toggle and remove the boundaries for the county and the communities? The icon to change that is visible on the screen right now. *(If it is not intuitive after 15 seconds of looking for the layer icon in the upper righthand corner* I will instruct them where to look).
- Can you access the **Groundwater Depth** information Icon and read the difference between **Cumulative** and **Discrete** bins

**Groundwater Hazard Checklist:**

- Convert from meters to feet
- Select 6.6 feet SLR
- Select 16.4 feet or shallower-cumulative
- Identify **Moderate Permeability**
- *circle back* set to **More Permeable**
- Toggle boundaries on and off

**Questions:**

1. Do the information ⓘ icons explain the difference between cumulative and discrete well? What do you think about the text that is on the pop-up

**Assets**

- *If they are struggling to identify and select **Improved Lands** in the **Economy** section within 5 minutes* Instruct them to navigate to the correct location in the asset dropdown in the appropriate place.

**Mini Task:**

- “Please pull up the information Icon under Assets and read the description of this feature”

**Question:**

1. Based on the organization of the **Assets** features, can you describe your thought-process for finding the appropriate selection. Did you expect to find Improved lands under the economy drop-down? (In my experience I expected it to be located under land types)
2. What do you observe with visualized polygons representing the Improved lands? What can you or can you not discern from the layover polygons on the map?

**Dashboard**

**Scenario #1**

- *If they cannot download data,* record where they are looking for the download feature. After a minute, instruct them to use the View Data Table tab and click the Community Data.

**Scenario #2**

- *After a minute,* guide the user to the Compare Places (municipalities) tab in the dashboard to find the bar graphs.
- Determine if they can produce the same numbers from the following

**Moderate Permeability**

**Aptos: $2.2M**

**Santa Cruz: $1.1B**

More Permeable

**Aptos: $720k**

**Santa Cruz: $950M**

**Scenario #3**

- *If they cannot get county level data to show up within a minute,* make sure the slider is set at County Scale to start if it glitches and says there is no data available
- Guide them to use the Scale County/Places slider then compare Places (municipalities) within the county using the Compare feature tab. See if they can determine the most impacted municipality in Santa Cruz County
- **Capitola at $77M under 16.4 feet groundwater depth**
- **Capitola at $57 under 6.6 feet groundwater depth**

**Plan B** (in the case that the user’s computer is lagging or that HERA crashes or will not load – and the persists for more than a few minutes – these set of questions will be asked) [5-7 minutes]

1. Based on your first impressions and navigation of the tool, how do you think the tool might be useful for the work you are doing?
a. Are rising groundwater hazards a concern in your work?
2. What other tools do you currently use to model or map similar outcomes?
a. What do you like about those tools
b. What gaps exist with your current tools
3. What do you find to be the most frustrating aspects of the HERA tool?
a. What improvements might you suggest?
4. and what parts do you enjoy the most in terms of the layout, colors, and text?
a. Do you have any suggestions?

**Error codes:**

- Requested data for selected places failed. Reduce the number of selected places and try again
- Ajax code
- Page unresponsive

**Hera Shoreline Change Usability Protocol**

**Introduction** [1 minute]

Briefly introduce yourself, the product, and the structure of the interview. Thank you for joining me to test the HERA tool. We scheduled this interview for 30 minutes but it may not take all that time. Is it alright if we record this interview for internal note-taking purposes? Thank you.

**[Press record]**

**Pre-test questions**[1.5 minutes]**:** Before we get started with the usability testing I would like to get a little bit of background information. These questions should only take a minute or two to answer before we begin the testing.

1. What is your current role at your organization?
2. What geography are you working on, and where are you located?
3. Who do you think of as the constituents or stakeholders that you collaborate with or work for?
4. How do you define the role of your work? For instance, do you think of yourself as a researcher, decision maker, or connector/facilitator?
5. What is your level of familiarity with HERA overall?
a. None: I’ve never heard of it
b. Very Low: I’ve heard of it, or seen it presented, but never used it myself
c. Low: I’ve heard of it, and tried it out at least once
d. Moderate: I am an occasional user (e.g., a few times a year, or periodically as a project might require)
e. Moderate-High: I’m a frequent user (e.g., a few times a month)
f. High: I use it all the time (e.g., weekly)
6. Ask same “level of familiarity” with the specific tool being tested (Groundwater or Shoreline Change)
7. What is your level of familiarity with the Coastal Storm Modeling System (CoSMoS), which provides the hazard exposure maps that underlie HERA?
a. None: I’ve never heard of it
b. Aware: I’ve heard of it, or seen it used, but I’ve never worked on a project where it was used
c. Familiar: I’ve worked on or reviewed a project/effort that has used the CoSMoS data or Our Coast Our Future viewer
d. Well-versed: I’ve worked on or reviewed multiple projects/efforts that have used the CoSMoS data or Our Coast Our Future viewer
e. Power user: I use it frequently (e.g., monthly) either directly with the GIS data or via the Our Coast Our Future viewer

**Script**

In a moment I’m going to put a link in the chat to our website. You may know some information about the site and the Hazards Exposure and Reporting Analytics (HERA) work, but we don’t want to give you too much background about it because we want to see how you use it. We’d like your feedback on anything from the way it looks to the way it functions. To gather this information from you, we’d like to spend the rest of our time doing what is referred to as a contextual inquiry. To do this, we ask that you pull up the link from the chat into a browser and share your screen with us so we can see how you interact with the site. As you explore the site, we ask that you talk out loud and walk me through what you’re thinking, and doing, and why. The goal of this discussion is to identify ways to improve users’ experience, so please do not hesitate to tell us any issues or things you think could make this a better site. Remember that this is NOT a test of your knowledge or abilities, but of the strengths and weaknesses of the HERA product design and delivery, so please don’t worry if you get stuck somewhere or if you “break” something.

**[Put website link in chat]**

<https://www.usgs.gov/apps/hera/shorelineChangeTool.php>

**Free scroll – 3 minutes**

What do you think this tool does?

**Usability Tasks** [25 minutes]

I am going to provide you with directions to complete a brief task with the HERA tool. The directions that I will provide will not be exhaustive so that we can test the user experience of the HERA’s design, not the user’s, or your, abilities. The end goal of this task is to determine the number of Occupied Housing Units in Miami Florida that are potentially at risk from coastal erosion due to sea level rise under the following conditions.

**[Read aloud then Paste these directions into the chat]**

- First, try to view results that show which communities in Miami-Dade County, Florida, have the highest number of Occupied Housing Units exposed to shoreline change hazards where **3.3 feet** of sea level rise, assuming that Beach Nourishment is **Continued**, the Existing Shoreline is **Maintained**, and Extreme Storm Erosion is I**ncluded**. If you can complete this task, please view the data in the dashboard
- Under these conditions for Miami-Dade County, which Communities are most impacted? How would you go about making this comparison?
- ***Once they have navigated to the Dashboard insert into the chat,*** If you were interested in the sum total of Occupied Housing for the entire County, how would you determine that? Can you shift the Dashboard to display statistics from specific Communities to the County level?
- If you were interested in comparing results from Miami-Dade County to Palm Beach County, how would you go about doing that?

**[If they complete the first scenario within 20 minutes, paste the second scenario into the chat]**

- Second, can you view results showing the number of Occupied Housing Units in Miami-Dade Florida exposed to shoreline change hazards where sea level rise is set at **9.8 feet**, Beach Nourishment is **Not Continued**, Existing Shoreline is **Not Maintained**, and Extreme Storm Erosion is **Included**. If you can complete this analysis, please view the results in the dashboard.
- Under these conditions for Miami-Dade County, which Communities are most impacted? How would you go about making this comparison?

**Troubleshooting and Questions to Ask**

**Places**

- *If you are struggling to select the correct geography*, In the tool, please go to the panel on the left and select Florida under **Places,** then unselect. A border should have popped up around the communities on the eastern coast.  After that, Follow the drop-down under Florida, and navigate to the county level, select Miami-Dade County and then navigate the drop-down in the county to only Miami, and select it.
- If they get stuck (or they cannot find the selection direct them to the ⓘ).

**Questions:**

- What is your impression of the overall aesthetics of the map component?
- Potential follow up/more specific prompts: Please describe what you are seeing on the map in terms of the colors, shapes, and contrast of various map layers. How do you feel about the ease/difficulty deciphering the different map layers?
- In terms of the response time of the application, are you noticing any lag at all while navigating between different areas or zooming-in or out?

**Sea Level Change Scenario**

**Mini Tasks:**

- 1) *If they get stuck (or when they cannot complete the correct selection, direct them to the ⓘ)*. Can you briefly talk through if this makes sense?
- 2) “How might you change the underlying basemap from the default satellite image to dark mode or street view?” The icon to change that is visible on the screen right now. *(If it is not intuitive after 15 seconds of looking for the layer icon in the upper righthand corner* I will instruct them where to look).

- 3) “Can you Zoom into the shoreline in any particular place then pull up the Map Legend and reduce the opacity of the Shoreline Change Hazard Zone (the green strip)?

**[Paste #3  prompt into the chat ]**

- *If they do not convert the meters to feet within 5 minutes for the Sea level Rise option, instruct them how to? If they do, ask to describe their process for finding it (was it easy)?

**Questions:**

- What do you think about the description of SLR Scenarios  ⓘ?  Can you please repeat the process of briefly interpreting ⓘ icon for beach nourishment.
- *If they adjust between multiple SLR scenarios* Ask them if they noticed if the tool is lagging when changing between SLR scenarios* Did you notice any lagging between scenarios/ changing between

**Assets**

**Mini Task:**

- “Please pull up the Information Icon under Assets and read the description of this feature”

Checklist:

- Did they use the County/Places slider to compare Places (municipalities) within the county using the Compare feature
- Did they know how to get help
- What help useful?

**Troubleshooting**

- *If they can cannot locate the **Assets for occupied housing units within 2 minutes*,** direct them to the selection under the drop-down for residents then housing units, and select option for Occupied.
- If they cannot change the County/Places slider In the Dashboard, ask them to view the Information Icon first. Then Direct them.

Next, prompt them to see if they can change the slider from County to Places

- Finally: Describe how to navigate to the menu option of the View Data Table in the **Dashboard** and ask them to try and download the county level data with said selections.

**Questions:**

- In the **Dashboard**, read through the display of the four graphs from right to left: 1) Number of Occupied Housing Units in Hazard Zone, 2) Number of Occupied Housing Units in Hazard Zone as Beach Nourishment Changes, 3) Number of Occupied Housing Units in Hazard Zone as Shoreline Infrastructure Changes, and (4) Number of Occupied Housing Units in Hazard Zone as Extreme Storm Erosion Changes.
- Ask if they expected these results?
- Can they see them displayed on the map?
- Then, ask them to talk through the differences in the distribution across the different places.
- After completed, ask “if the categorization for occupied housing units made sense in the organization of the Asset selection.

**Wrap Up [questions if there is time, est. 2 minutes]**

1. Do you think that you would use this data in your current work if it was available for the region where you work? If so, how? If not, why not?
2. If available, do you think that you would use this site with your constituencies or partners? If not, why not?
3. What kinds of platforms/hardware would you use it on? Such as accessing HERA via desktop, laptop, or tablet.
4. Following up from the free scroll, what do you think that this tools main function is?

Thank you for your time. We will be speaking with several other intended users and will compile the results of all of the interviews. If you have any further questions or comments, please reach out to me at <your email here>.
